# Age- and sex-specific associations between risk scores for schizophrenia and self-reported health in the general population

**DOI:** 10.1007/s00127-022-02346-3

**Published:** 2022-08-01

**Authors:** Vincent Paquin, Lotta-Katrin Pries, Margreet ten Have, Maarten Bak, Nicole Gunther, Ron de Graaf, Saskia van Dorsselaer, Bochao D. Lin, Kristel R. van Eijk, Gunter Kenis, Alexander Richards, Michael C. O’Donovan, Jurjen J. Luykx, Bart P. F. Rutten, Jim van Os, Jai L. Shah, Sinan Guloksuz

**Affiliations:** 1 Prevention and Early Intervention Program for Psychosis (PEPP-Montreal), Douglas Mental Health University Institute, Montreal, QC Canada; 2 Department of Psychiatry, McGill University, Montreal, QC Canada; 3 Department of Psychiatry and Neuropsychology, School of Mental Health and Neuroscience, Maastricht University Medical Centre, Vijverdalseweg 1, SN.2.068, P.O.Box 616 6200, Maastricht, MD The Netherlands; 4 Department of Epidemiology, Netherlands Institute of Mental Health and Addiction, Utrecht, The Netherlands; 5 FACT, Mondriaan Mental Health, Maastricht, The Netherlands; 6 School of Psychology, Open University, Heerlen, The Netherlands; 7 Department of Psychiatry, UMC Utrecht Brain Center, University Medical Centre Utrecht, Utrecht, The Netherlands; 8 Research Institute Brainclinics, Brainclinics Foundation, Nijmegen, The Netherlands; 9 Department of Neurology, UMC Utrecht Brain Center, University Medical Centre Utrecht, Utrecht, The Netherlands; 10 Division of Psychological Medicine and Clinical Neurosciences, MRC Centre for Neuropsychiatric Genetics and Genomics, School of Medicine, Cardiff University, Cardiff, UK; 11 GGNet Mental Health, Warnsveld, The Netherlands; 12 Department of Psychosis Studies, Institute of Psychiatry, King’s College London, London, UK; 13 Department of Psychiatry, Yale University School of Medicine, New Haven, CT USA

**Keywords:** Exposome, Genes, Schizophrenia, Quality of life, Sex characteristics

## Abstract

**Purpose:**

The health correlates of polygenic risk (PRS-SCZ) and exposome (ES-SCZ) scores for schizophrenia may vary depending on age and sex. We aimed to examine age- and sex-specific associations of PRS-SCZ and ES-SCZ with self-reported health in the general population.

**Methods:**

Participants were from the population-based Netherlands Mental Health Survey and Incidence Study–2 (NEMESIS-2). Mental and physical health were measured with the 36-item Short Form Survey 4 times between 2007 and 2018. The PRS-SCZ and ES-SCZ were respectively calculated from common genetic variants and exposures (cannabis use, winter birth, hearing impairment, and five childhood adversity categories). Moderation by age and sex was examined in linear mixed models.

**Results:**

For PRS-SCZ and ES-SCZ analyses, we included 3099 and 6264 participants, respectively (age range 18–65 years; 55.7–56.1% female). Age and sex did not interact with PRS-SCZ. Age moderated the association between ES-SCZ and mental (interaction: *p* = 0.02) and physical health (*p* = 0.0007): at age 18, + 1.00 of ES-SCZ was associated with − 0.10 of mental health and − 0.08 of physical health, whereas at age 65, it was associated with − 0.21 and − 0.23, respectively (all units in standard deviations). Sex moderated the association between ES-SCZ and physical health (*p* < .0001): + 1.00 of ES-SCZ was associated with − 0.19 of physical health among female and − 0.11 among male individuals.

**Conclusion:**

There were larger associations between higher ES-SCZ and poorer health among female and older individuals. Accounting for these interactions may increase ES-SCZ precision and help uncover populational determinants of environmental influences on health.

**Supplementary Information:**

The online version contains supplementary material available at 10.1007/s00127-022-02346-3.

## Introduction

The polygenic risk score (PRS-SCZ) and exposome score for schizophrenia (ES-SCZ) are indices of liability to schizophrenia. The PRS-SCZ captures cumulative effects of common genetic variants on the risk for schizophrenia [1, 2], while the ES-SCZ accounts for a range of schizophrenia-associated exposures such as cannabis use, winter birth, hearing impairment, and childhood adversity [3, 4]. To date, the PRS-SCZ and ES-SCZ are among the most robust and validated polygenic and exposome scores for mental disorders. Although these risk indices were developed to study the populational liability to schizophrenia, both scores are further associated with other relevant health conditions: in the general population, in addition to schizophrenia spectrum disorders [5, 6], they are associated with common mental disorders and a range of physical health outcomes [5–8]. The utility of PRS-SCZ and ES-SCZ thus extends to studying shared pathways of risk between schizophrenia and other health outcomes.

However, the health correlates of PRS-SCZ and ES-SCZ may vary as a function of age and sex [9–11]. For example, in non-clinical samples, PRS-SCZ seems to be more strongly related to cognition during late life [12], and its associations with cognitive task performance and schizotypy may be male-specific [13, 14]. Age- and sex-specific associations have not been examined with ES-SCZ specifically, but the count of schizophrenia-associated exposures is associated with earlier age at onset of psychosis [15, 16], and cumulative exposure to childhood adversity is associated with greater risk for affective disorders and physical health problems among women [17, 18]. Together, these findings tentatively suggest that for both the PRS-SCZ and ES-SCZ, epidemiological associations with health measures may not be uniform across ages and sexes.

We thus aimed to examine whether age and sex moderate the associations between risk scores for schizophrenia and self-reported health in a general population-based cohort. We considered two risk scores (PRS-SCZ and ES-SCZ) and two outcomes (self-reported mental and physical health). We also aimed to explore whether the interactions between risk scores and age differed according to sex (three-way interactions).

## Materials and methods

### Participants

Participants were from the Netherlands Mental Health Survey and Incidence Study–2 (NEMESIS-2), which was designed to investigate the prevalence, incidence, course, and consequences of mental disorders in the Dutch general population [19, 20]. The study was approved by the Medical Ethics Review Committee for Institutions on Mental Health Care (METIGG). All participants provided written informed consent.

Participants were recruited according to a multistage random sampling procedure to ensure representativeness for age (between 18 and 65 years), region, and population density [19]. Individuals not proficient in Dutch were excluded. Participants were assessed by trained interviewers on four occasions between 2007 and 2018: at baseline (T0), at year 3 (T1), at year 6 (T2) and at year 9 (T3). The first wave (T0) included 6646 participants (response rate 65.1%; average interview duration: 95 min). Subsequent response rates were 80.4% at T1 (*N* = 5303; excluding those who deceased; average interview duration: 84 min), 87.8% at T2 (*N* = 4618; interview duration: 83 min), and 87.7% at T3 (*N* = 4007; interview duration: 101 min). Attrition between T0 and T3 was associated with younger age, lower educational attainment, unemployment, and being born outside the Netherlands [21]. There was no association of attrition with baseline 12-month common mental disorders (after adjusting for sociodemographic characteristics) nor with having any chronic physical disorder.

### Measures

#### Mental and physical health outcomes

Health outcomes were measured at T0, T1, T2, and T3 with the 36-item Short Form Survey (SF-36) [22]. The SF-36 includes 8 subscales, each ranging from poor (0) to good (100) health. Based on previous validation studies of the questionnaire in community-based and clinical samples [22, 23], we aggregated the subscales into 2 general measures of mental and physical health: (1) the “mental health”, “role limitations due to emotional problems”, “social functioning”, and “vitality” subscales were averaged into a single mental health score, and (2) the “general health”, “physical functioning”, “role limitation due to physical health problems”, and “bodily pain” subscales were averaged into a single physical health score.

#### Polygenic risk score for schizophrenia

Details on the genotyping procedure are presented in the Supplementary Material. For PRS-SCZ, we selected schizophrenia-associated genetic loci according to a P-threshold of < 0.05 because this threshold best captures liability to the disorder according to the Psychiatric Genomics Consortium analysis [24]. With the same cohort and measures as the current study, we previously reported that higher PRS-SCZ was significantly associated with poorer mental health, but not significantly associated with physical health; these associations did not account for potential interactions with age or sex [8].

#### Exposome score for schizophrenia

For ES-SCZ, we selected eight exposures from T0 which we aggregated following a previously validated method [3]. These exposures were cannabis use, winter birth, hearing impairment, and five domains of childhood adversity. Cannabis use was defined as one use per week or more in the period of most frequent use (lifetime) and was measured with the Illegal Substance Use section of the Composite International Diagnostic Interview 3.0 [25, 26]. Winter birth was defined as birth between December and March. Hearing impairment was reported by participants for the past 12 months. Participant reports of childhood adversity were collected with the modified NEMESIS-1 trauma questionnaire [19] and were divided in five domains: emotional abuse, physical abuse, sexual abuse, emotional neglect, and bullying. We coded all exposures as present = 1 or absent = 0. ES-SCZ was calculated by summing these exposures, each weighted by their schizophrenia-associated log odds in an external sample (total range 0–5.95, with higher values indicating higher risk for schizophrenia) [3]. With the same cohort and measures as the current study, we previously reported that higher ES-SCZ was significantly associated with poorer mental and physical health [8], but these associations did not account for potential interactions with age or sex.

### Statistical analysis

Analyses were conducted in R version 3.6.3 (R Foundation for Statistical Computing). We defined nominal statistical significance as *p* < 0.05. We excluded participants who had missing data on modeled variables at baseline; those with complete baseline data were included even if they were subsequently lost to follow-up. To evaluate the potential impact of excluding participants on our analyses, we compared sociodemographic characteristics of included participants with those of excluded participants.

To examine age- and sex-specific associations between “risk” scores and mental health or physical health outcomes, we applied linear mixed models using the *nlme* package [27]. Rather than focusing on cross-sectional measures of mental and physical health at T0, we analyzed the repeated measures of these outcomes over time to capture some of their within-individual variance. Of note, linear mixed models are robust to attrition over follow-up if missingness is at random. Due to the temporal dependency between outcome measures, we applied an autoregressive correlation structure of order 1 [27]. We adjusted for the timing of outcome ranging from 0–9 years since T0. In analyses of age as the moderator, we included age fixed at T0 as a time-invariant predictor spanning 18 through 65 years of age, the risk score (either PRS-SCZ or ES-SCZ), and the interaction between the risk score and age at T0. For all analyses of age, quadratic effects of age and their interaction with risk scores improved model fit by a reduction of ≥ 10 of the Akaike Information Criterion and were thus included [28]. In analyses of sex as the moderator, we included a dichotomous variable for sex, the risk score, and the interaction between the risk score and sex. We standardized (mean = 0, SD = 1) the PRS-SCZ, ES-SCZ, and outcome variables. We divided age by 10 to reduce its scaling difference relative to other variables (but transformed it back to its original scale in the figures). In PRS-SCZ models, we adjusted for the first three principal components which we also standardized. We probed interaction effects by estimating the marginal trend of outcome associated with 1 SD increase in the risk score at different levels of the moderator (age or sex) [29].

When interactions between ES-SCZ and age or sex were statistically significant, we conducted a sensitivity analysis to explore their stability. We did so by testing the same interactions but using eight variations of the ES-SCZ, each omitting one of the 8 schizophrenia-associated exposures. Lastly, to explore whether interactions between PRS-SCZ or ES-SCZ and age differed between participants of male and female sexes, we tested three-way interactions between the risk scores, age, and sex.

## Results

### Interactions between the polygenic risk score for schizophrenia and age or sex

Of 6646 participants enrolled in NEMESIS-2, 3099 (46.6%) had complete baseline data for analyses involving the PRS-SCZ and were thus included in these analyses. Included participants had a mean (SD) age of 44.1 (12.5) years and 56.1% were female. As shown in Table 1, included participants were more likely to have higher educational attainment compared with excluded participants, but they did not significantly differ on other characteristics.

**Table 1:** Baseline characteristics of participants in the Netherlands Mental Health Survey and Incidence Study–2

	Polygenic analyses			Exposome analyses		
	Included participants (*N* = 3099)	Excluded participants (*N* = 3547)	*p* value	Included participants (*N* = 6264)	Excluded participants (*N* = 382)	*p* value
Sex, *N* (%):			0.21			0.002
Male	1361 (43.9%)	1613 (45.5%)		2773 (44.3%)	201 (52.6%)	
Female	1738 (56.1%)	1934 (54.5%)		3491 (55.7%)	181 (47.4%)	
Education, *N* (%):			< 0.001			< 0.001
Primary school	128 (4.13%)	204 (5.75%)		288 (4.60%)	44 (11.5%)	
Lower secondary	799 (25.8%)	1027 (29.0%)		1732 (27.7%)	94 (24.6%)	
Higher secondary	1017 (32.8%)	1128 (31.8%)		2001 (31.9%)	144 (37.7%)	
Higher professional	1155 (37.3%)	1188 (33.5%)		2243 (35.8%)	100 (26.2%)	
Age in years, mean (SD)	44.1 (12.5)	44.4 (12.5)	0.48	44.5 (12.4)	40.1 (13.5)	< 0.001
Mental health, SF-36, mean (SD)	85.2 (13.4)	84.5 (14.4)	0.08	85.0 (13.8)	81.2 (16.2)	< 0.001
Physical health, SF-36, mean (SD)	84.3 (17.3)	83.9 (18.0)	0.42	84.2 (17.6)	82.7 (18.7)	0.047

*P* values for group comparisons are from chi-squared, exact Fisher, and Kruskall-Wallis tests

*SD* standard deviation, *SF-36* 36-item Short Form Survey

**Fig. 1:**
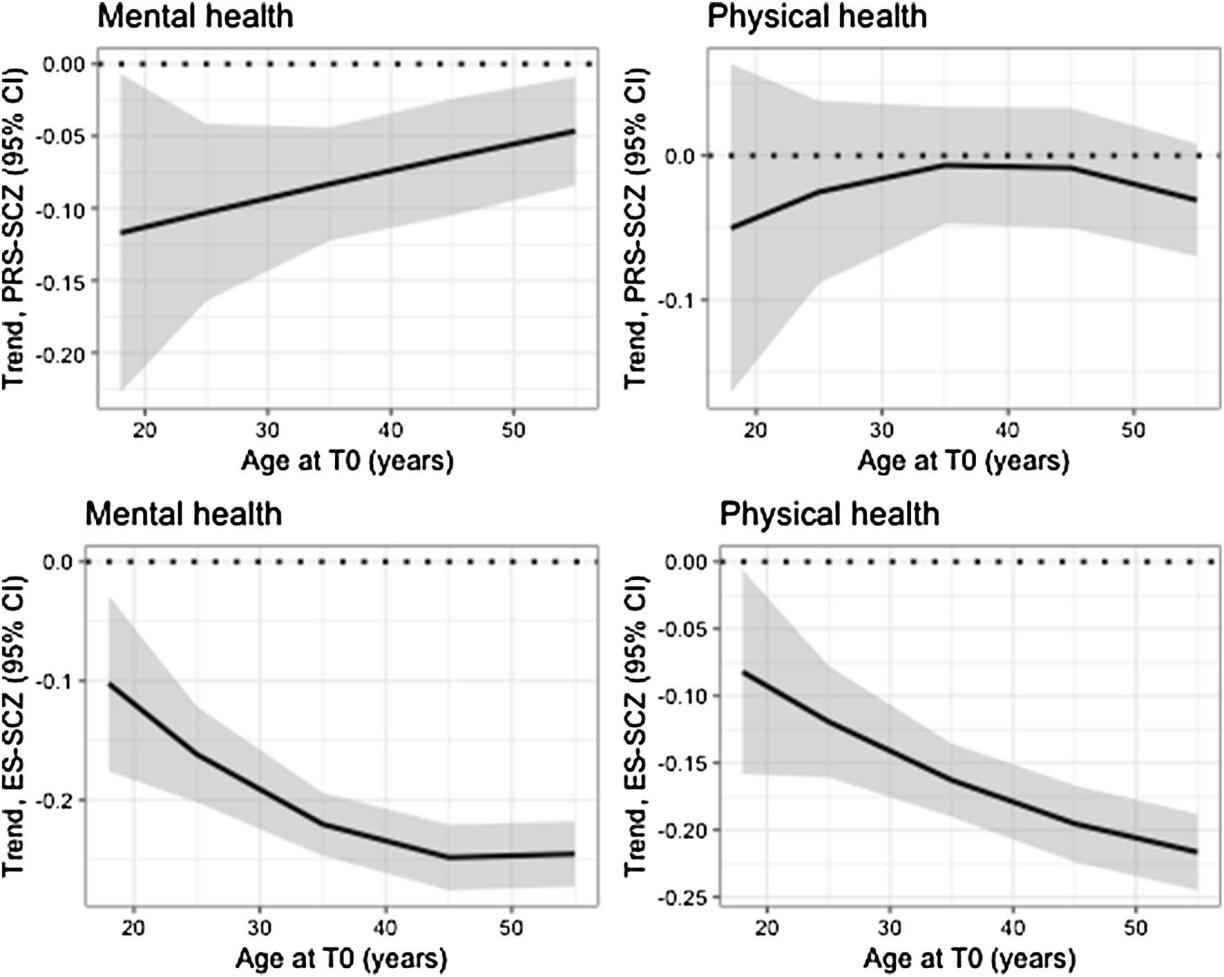
Interactions between schizophrenia risk scores and age associated with mental health or physical health. PRS-SCZ indicates polygenic risk score for schizophrenia (upper row). ES-SCZ indicates exposome score for schizophrenia (lower row). Outcomes were mental health (on the left) and physical health (on the right) as measured with the 36-item Short Form Survey. Using linear mixed models, we estimated the trend between PRS-SCZ or ES-SCZ and the outcomes at various ages, including linear and quadratic effects of age on these trends

**Fig. 2:**
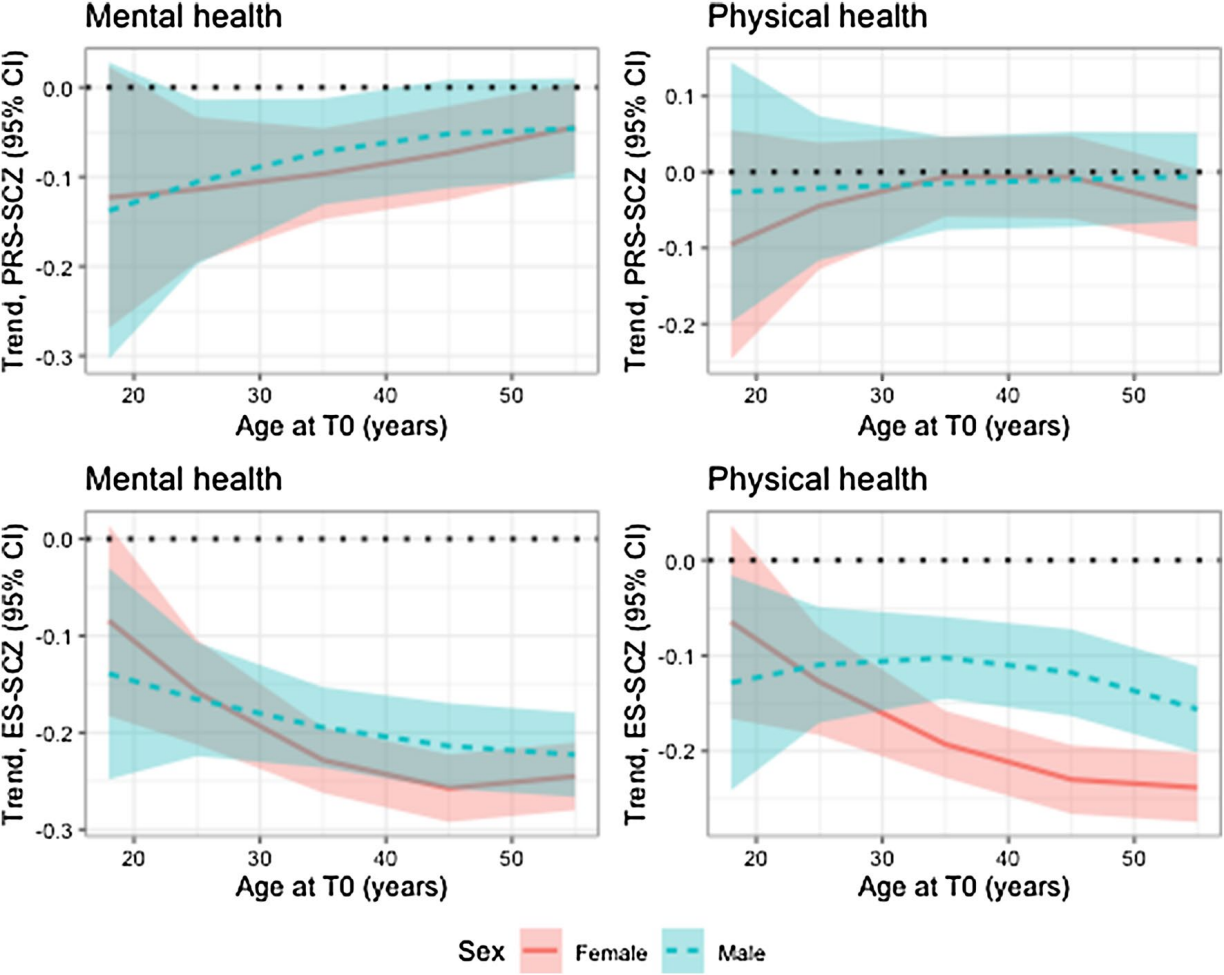
Three-way interactions between schizophrenia risk scores, age and sex associated with mental health or physical health. PRS-SCZ indicates polygenic risk score for schizophrenia (upper row). ES-SCZ indicates environmental risk score for schizophrenia (lower row). Outcomes were mental health (on the left) and physical health (on the right) as measured with the 36-item Short Form Survey. Using linear mixed models, we estimated the trend between PRS-SCZ or ES-SCZ and the outcomes at various ages, including linear and quadratic effects of age on these trends, and according to sex

Table 2 presents the PRS-SCZ × age and PRS-SCZ × sex interaction terms. For both the mental health and physical health outcomes, there was no significant interaction of PRS-SCZ with age or sex. Figure 1 (upper row) illustrates the associations between PRS-SCZ and outcomes according to the age of participants. The trends on Fig. 1 suggest that PRS-SCZ was associated with poorer mental health, particularly at younger ages, and poorer physical health, particularly at the “extremes” of the age interval (18–65 years); however, as illustrated by the confidence intervals that largely overlap across ages, these age-specific patterns were not statistically significant for both mental and physical health. Supplementary Table 1 presents associations between PRS-SCZ and outcomes according to sex. Sex did not moderate the interactions between PRS-SCZ and age (Fig. 2).

**Table 2:** Coefficients of interactions between polygenic risk score for schizophrenia and age or sex

Outcome	PRS-SCZ × age				PRS-SCZ × sex	
	Coefficient, with linear age (95% CI)	*p* value	Coefficient, with quadratic age (95% CI)	*p* value	Coefficient (95% CI)	*p* value
Mental health	2.31 (− 0.53, 5.16)	0.11	− 0.07 (− 2.98, 2.85)	0.96	− 0.01 (− 0.06, 0.05)	0.78
Physical health	− 1.04 (− 3.97, 1.89)	0.49	− 1.60 (− 4.60, 1.41)	0.30	− 0.03 (− 0.09, 0.03)	0.26

Linear mixed models of mental health and physical health scores as measured with the 36-item Short Form Survey. PRS-SCZ indicates polygenic risk score for schizophrenia. Age is at baseline (T0) in years divided by 10. All models are adjusted for time of outcome since T0 and the first three principal components

### Interactions between the exposome score for schizophrenia and age or sex

Of the total cohort, 6264 participants (94.2%) had complete baseline data for analyses involving the ES-SCZ. Included participants had a mean (SD) age of 44.5 (12.4) years and 55.7% were female. As shown in Table 1, included participants were more likely to be female and to have higher educational attainment compared with excluded participants. Inclusion was also associated with higher age and higher scores for mental health and physical health.

Coefficients for interactions between ES-SCZ and age or sex are presented in Table 3. For mental health, there was a significant interaction between ES-SCZ and age (through both its linear and quadratic terms). As shown in Fig. 1 (bottom left), higher ES-SCZ was significantly associated with lower mental health levels, and this negative association was of greater magnitude among older participants. To illustrate, at age 18, higher ES-SCZ by 1.00 SD was associated with lower mental health by 0.10 SD, whereas at age 65, the same increase in ES-SCZ was associated with lower mental health by 0.21 SD. The interaction between ES-SCZ and age had a similar pattern in all 8 sensitivity analysis models using variations of the ES-SCZ (Supplementary Fig. 1). Across these models, the interaction between ES-SCZ and age, either through its linear or quadratic term, was significant (Supplementary Table 2). There was no interaction between ES-SCZ and sex (Table 3, Supplementary Table 1). The three-way interaction between ES-SCZ, age, and sex was not significant (Fig. 2): coefficient (with linear age) = 0.11 (95% CI − 5.45, 5.67), *p* = 0.97, and coefficient (with quadratic age) = 3.35 (95% CI − 2.31, 9.02), *p* = 0.25.

**Table 3:** Coefficients of interactions between exposome score for schizophrenia and age or sex

Outcome	ES-SCZ × age				ES-SCZ × sex	
	Coefficient, with linear age (95% CI)	*p* value	Coefficient, with quadratic age (95% CI)	*p* value	Coefficient (95% CI)	*p* value
Mental health	− 3.00 (− 5.75, − 0.24)	0.03	3.31 (0.51, 6.10)	0.02	− 0.03 (− 0.01, 0.01)	0.19
Physical health	− 4.93 (− 7.79, − 2.07)	0.0007	1.20 (− 1.70, 4.09)	0.42	− 0.09 (− 0.13, − 0.04)	< 0.0001

Linear mixed models of mental health and physical health scores as measured with the 36-item Short Form Survey. ES-SCZ indicates exposome score for schizophrenia. Age is at baseline (T0) in years divided by 10. All models are adjusted for time of outcome since T0

For physical health, there was a significant interaction between ES-SCZ and the linear term for age (Table 3). Higher ES-SCZ was significantly associated with lower physical health levels, and this association was of greater magnitude among older participants (Fig. 1, bottom right). At age 18, higher ES-SCZ by 1.00 SD was associated with lower physical health by 0.08 SD; at age 65, higher ES-SCZ by 1.00 SD was associated with lower physical health by 0.23 SD. The interaction between ES-SCZ and linear age remained significant across variations of the ES-SCZ after alternately omitting individual exposures from ES-SCZ; one exception was after omitting cannabis use (interaction coefficient: *p* = 0.09; Supplementary Table 3). A consistent interaction pattern was nonetheless identified in all 8 sensitivity analysis models, including the one omitting cannabis use (Supplementary Fig. 2). Next, there was a significant interaction between ES-SCZ and sex (Table 3). Higher ES-SCZ was associated with lower physical health, and this association was of greater magnitude among female participants (Supplementary Table 1): in female participants, higher ES-SCZ by 1.00 SD was associated with lower physical health by 0.19 SD, whereas in male participants, higher ES-SCZ by 1.00 SD was associated with lower physical health by only 0.11 SD. In sensitivity analyses, this interaction remained significant and was in the same direction for all 8 models (Supplementary Table 4). The three-way interaction between ES-SCZ, age, and sex was not nominally significant: coefficient (with linear age) = − 0.70 (95% CI − 6.45, 5.04), *p* = 0.81, and coefficient (with quadratic age) = 5.52 (95% CI − 0.33, 11.38), *p* = 0.06. However, visual probing of the interaction (Fig. 2, bottom right) suggests that the interaction between ES-SCZ and age (i.e., a greater association between ES-SCZ and physical health as a function of older age) was specific to female individuals.

## Discussion

The association between exposomic liability to schizophrenia and poorer mental and physical health was greater with older age. To our knowledge, this is the first study to explore whether exposomic liability to schizophrenia has age-specific associations with health. One explanation for this finding is that the health influences of schizophrenia-associated exposures may accumulate over the lifespan. In NEMESIS-2, we previously found that ES-SCZ was associated with multiple mental and physical conditions, such as social phobia, asthma, joint wear and heart disease [5], which typically emerge at various ages. The age-dependent trends of ES-SCZ may thus reflect cumulative incidence of health conditions throughout adulthood.

The association between ES-SCZ and physical health was also greater among female individuals. Sex or gender differences are often identified in the associations between risk factors for schizophrenia and health, although sometimes inconsistently. To illustrate, in a survey of middle-aged adults, childhood adversity was preferentially associated with obesity, hypertension, and cardiovascular disease among men, and with insomnia and cancer among women [30]. Another study found the opposite pattern, concluding that childhood adversity was more strongly associated with heart disease in women [17]. Cannabis use, childhood adversity, and other exposures have displayed sex-specific associations with mental health in previous research [31, 32], yet we observed an interaction with sex for physical health only, and not for mental health. Ultimately, mechanisms for these sex differences are complex and may include multiple social and environmental factors, genes, neurodevelopmental trajectories, immune responses, and gonadal hormones, and the relative contribution of these mechanisms may be heterogeneous depending on population characteristics, measurements, and outcome selection [11, 31].

The associations between PRS-SCZ and health measures did not interact with age or sex. Genetic variants conferring risk for schizophrenia have been associated with multiple mental and physical health outcomes [6, 33, 34]: for example, in an electronic health record study of 106,160 adults in the US, higher PRS-SCZ was associated with higher risk of mood and anxiety disorders, neurological conditions, and urinary syndromes, as well as lower risk of synovitis and obesity [6]. There is however limited literature on interactions between PRS-SCZ and age. Perhaps the study most comparable to the current analysis comes from a longitudinal cohort, where PRS-SCZ was associated with IQ at age 70, but not at age 11 [12]. Considering that neurodevelopment may underlie shifts in the correlates of PRS-SCZ between ages of 11 and 70, it is unclear if a similar interaction would apply to the age range of the current study (18–65 years old), and if it would extend to the broader health measures we examined. As for genetic heterogeneity by sex, a recent genome-wide analysis study of schizophrenia found no evidence thereof [35]. Another study found evidence of greater expression of schizophrenia-associated genes in male compared with female brains [36]. Further, the PRS-SCZ has displayed male-specific associations with neural connectivity [37], schizotypy [14], and cognitive task performance [13] in other work. Our negative findings may reflect a true absence of interaction between PRS-SCZ and age or sex, but they may also be due to insufficient statistical power. As mentioned in the introduction, the association between PRS-SCZ and health in the general population is weak at best, and statistical power decreases dramatically for interaction effects of the same magnitude [38]. Thus, our sample size and the PRS-SCZ’s predictive performance may still be insufficient to reliably detect interactions.

Overall, we provide preliminary evidence of age- and sex-specific associations between ES-SCZ and self-reported health in the general population. As these findings and the other studies cited above illustrate, risk factors for schizophrenia have transdiagnostic correlates. They are not only associated with schizophrenia risk, but also with other mental and physical health outcomes across the lifespan [5, 6, 8]. In recent years, the ES-SCZ has been combined with other predictive variables to study the risk architecture of symptom profiles, illness status, and health outcomes across clinical and non-clinical samples [4]. By aggregating exposures associated with the liability to schizophrenia, the ES-SCZ offers a practical approach to capturing the additive and interdependent effects of multiple exposures on health. Alongside other clinical and biological data, exposomic liability to schizophrenia may help advance the personalization of early intervention services–not only as an index of psychosis risk, but also of other diagnostic categories and domains of health [39]. Yet while environmental exposures have sizeable health correlates, these properties may be highly contingent on other factors, from the biological to the social. Appreciating interactions between exposome scores and sociodemographic factors, including age and sex, should thus be considered for the scores to perform optimally across various populations, and to increase our understanding of the pathways underlying non-specific exposomic associations with health.

### Strengths and limitations

A strength of the study is that we examined exposomic liability to schizophrenia in a large sample drawn from the general Dutch population. Not surprisingly given the sample size, there were statistically significant differences in baseline characteristics after excluding participants with missing data. But considering the relatively small magnitude of differences, external validity may still be reasonable for the target population [19]. For genetic analyses, however, the representativeness of our sample was limited: we excluded individuals of non-European ancestries because the PRS-SCZ we used is only valid for populations of European ancestry [2].

We analyzed interactions with age according to between-individual rather than within-individual differences in age because participants were followed over less than a decade. The advantage of this is that the wide age interval of participants at baseline (18–65 years old) allowed us to capture moderation by age across most of the adult life period. The limitation of this approach is that it prevented us from differentiating interactions with age (that would arise from longitudinal health changes within individuals as they age) from interactions with the period of birth. The health correlates of ES-SCZ may vary as a function of the period of birth due to historical factors [40–42]. Here, study participants aged 18 years at baseline (2007–2009) were born in 1989–1991, while those aged 65 were born in 1942–1944. Although the time span was relatively short, we cannot exclude a possible contribution of generational differences to the interaction between ES-SCZ and age.

Measures included sex but not gender, and both constructs are relevant to untangle the social and biological factors that may play a role in moderating the association between risk scores for schizophrenia and health. As for outcomes, we benefitted from repeated measures over time to increase the precision of the statistical models. We focused on self-reported mental and physical health, two broad constructs that are meaningful across the lifespan in the general population. But to develop a more granular understanding of interactions between ES-SCZ or PRS-SCZ and age and sex, further work should examine the incidence of specific clinical outcomes during relevant age periods (e.g., cardiac disease from mid-adulthood onwards).

Finally, due to the challenges of identifying and validating consistent measures of exposures across distinct samples, ES-SCZ is composed of only a handful of the risk factors for schizophrenia; inclusion of additional exposures, such as perinatal adversity [11], could increase its predictive performance and influence its interactions with age and sex. But while the ES-SCZ is calculated from only eight exposures, its interactions with age and sex were largely robust to omitting single exposures, suggesting relatively stable age- and sex-specific trends. The interaction between ES-SCZ and age was also consistent across both mental and physical health, further supporting the robustness of this finding.

In conclusion, we found that ES-SCZ, but not PRS-SCZ, interacted with age and sex in association with self-reported health in a Dutch population-based cohort. Our findings support the relevance of considering whether population characteristics moderate the transdiagnostic associations between exposome scores and health.

## Supplementary Information

Below is the link to the electronic supplementary material.

Supplementary file1 (DOCX 635 KB)

## Data Availability

The data on which this manuscript is based are not publicly available. However, data from NEMESIS-2 are available upon request. The Dutch ministry of health financed the data and the agreement is that these data can be used freely under certain restrictions and always under supervision of the principal investigator (PI) of the study. Thus, some access restrictions do apply to the data. At any time, researchers can contact the PI of NEMESIS-2 and submit a research plan, describing its background, research questions, variables to be used in the analyses, and an outline of the analyses. If a request for data sharing is approved, a written agreement will be signed stating that the data will only be used for addressing the agreed research questions described and not for other purposes.
