# Supplementary material for: Age- and sex-specific associations between risk scores for schizophrenia and self-reported health in the general population
Source: Soc Psychiatry Psychiatr Epidemiol. 2022 Aug 1;58(1):43–52. doi: 10.1007/s00127-022-02346-3 (PMC9845157; doi:10.1007/s00127-022-02346-3)
Supplement: Supplementary file 1 — Supplementary file1 (DOCX 635 KB) [file 127_2022_2346_MOESM1_ESM.docx]

Table of Contents

[Supplementary Methods: Genotyping procedures, quality control, imputation, and polygenic risk score (PRS) calculation 2](#_Toc94950035)

[Supplementary Table 1. Interactions between schizophrenia risk scores and sex 8](#_Toc94950036)

[Supplementary Table 2. Sensitivity analysis of the interaction between ES-SCZ and age in association with mental health 9](#_Toc94950037)

[Supplementary Table 3. Sensitivity analysis of the interaction between ES-SCZ and age in association with physical health 10](#_Toc94950038)

[Supplementary Table 4: Sensitivity analysis of the interaction between ES-SCZ and sex in association with physical health 11](#_Toc94950039)

[Supplementary Figure 1. Sensitivity analysis of the interaction between ES-SCZ and age in association with mental health 12](#_Toc94950040)

[Supplementary Figure 2. Sensitivity analysis of the interaction between ES-SCZ and age in association with physical health 13](#_Toc94950041)

[References 14](#_Toc94950042)

# **Supplementary Methods: Genotyping procedures, quality control, imputation, and polygenic risk score (PRS) calculation**

***1. Target Genotype Data Processing***

*1.1. Genotyping procedures and quality control steps before imputation*

NEMESIS-2 samples were genotyped on an IPMCN chip (Institute of Psychological Medicine and Clinical Neurology, Cardiff University), which was custom-made for EUGEI (588,628 genotyped variants for 4,043 participants).^1^ Quality control (QC) was done using PLINK v1.9^2^ as follows. There were 3,861 samples matching with phenotypes. Single nucleotide polymorphisms (SNPs) and samples with call rates below 95% and 98% respectively were removed. A strict SNP QC only for subsequent sample QC steps was conducted. This involved a minor allele frequency (MAF) threshold >10% and a Hardy-Weinberg equilibrium (HWE) P-value >10^-5^, followed by linkage disequilibrium (LD) based SNP pruning (R^2^ < 0.5). This resulted in ~60K SNPs to assess sex errors (n=145), heterozygosity (F< 5xSD the standard deviation (SD), n= 73), and relatedness by pairwise identity by descent (IBD) values > 0.1 (n= 170). Genetic outliers (n=154) were identified by principal component analysis (PCA, see below). In total, 3104 individuals passed these QC steps. After removing failing samples (n = 757), a regular SNP QC was performed (SNP call rate>95%, HWE p>1e-06, MAF> 0.16%; as the IPMCN chip contains many rare variants, half the SNPs would have been removed if we had applied MAF> 1%; therefore, we loosened MAF threshold to 0.16% =10/(2*sample size of 3,104)). Next, strand ambiguous SNPs and duplicate SNPs were removed, resulting in a total of 298,104 genotyped variants.

*1.2. Imputation on Michigan server.*

The QC-ed dataset was chunked by chromosome, and then converted into *.VCF files. The Michigan server was used for imputation with the following settings: reference panel as HRC R1.1 2016; phasing as Eagle v2.3; population as European; model as QC & imputation. The imputation resulted in 47,101,073 single nucleotide polymorphisms (SNPs). The general imputation quality is shown in Figure I.

*Figure I. Correlation of SNPs MAF from NEMESIS-2 dataset with the reference MAF.*

*
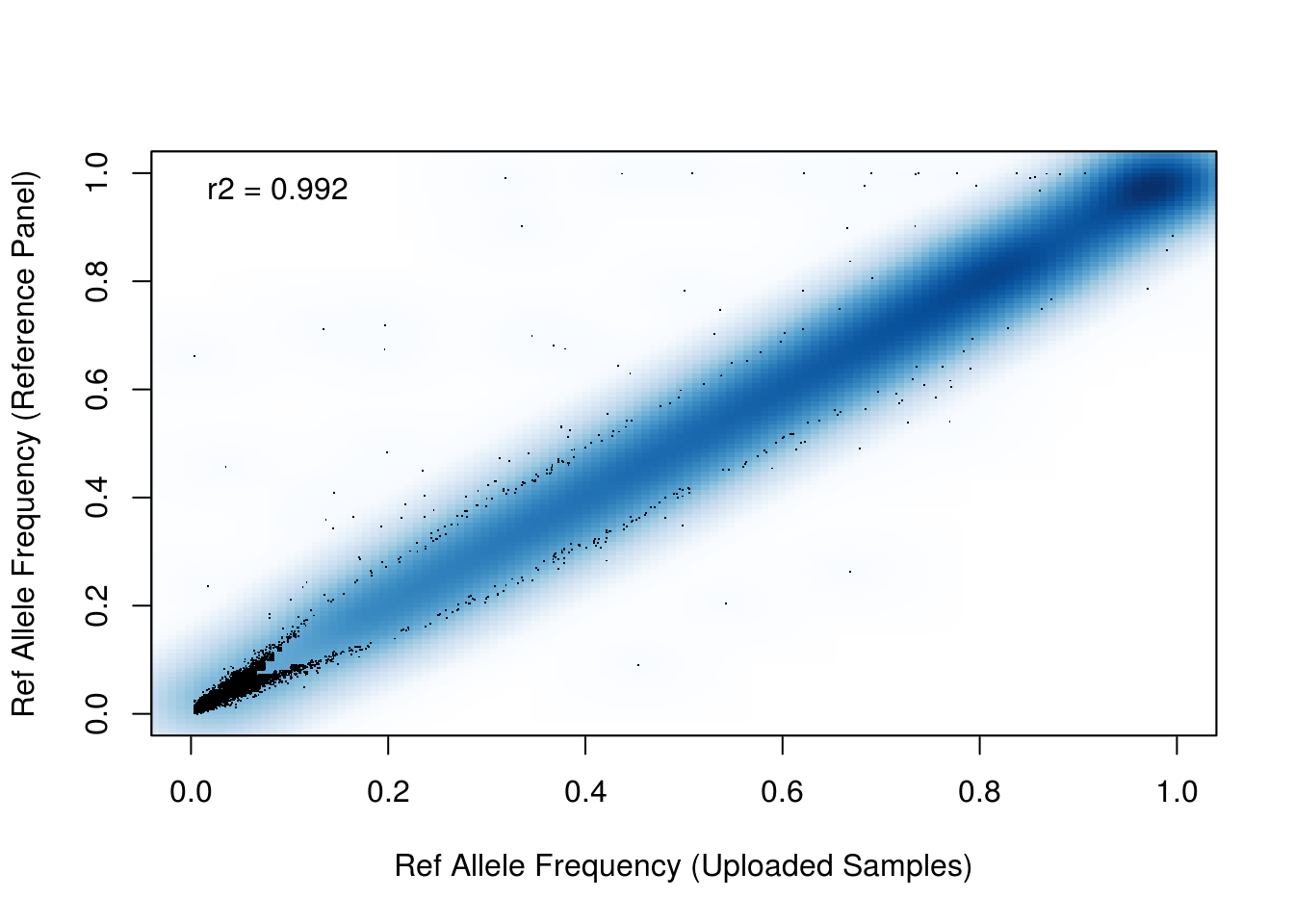
*

*1.3. Quality control after imputation.*

Poor quality SNPs were excluded: multi-allelic SNPs, SNPs with a minor allele frequency (MAF)<0.0016 or INFO<0.3, and strand ambiguous AT/CG SNPs. The VCF dosage files were converted into PLINK format dosage files. Finally, 10,356,437 SNPs and 3,104 individuals remained. For hard call (best guess) genotypes, an additional SNP QC (INFO>0.8 and HWE>10-6) was performed, resulting in 6,436,459 SNPs.

*1.4. Principle components analyses (PCA).*

The principal components (PCs) analyses within NEMESIS-2 samples, and NEMESIS-2 samples along with Hapmap3 (<https://www.sanger.ac.uk/resources/downloads/human/hapmap3.html>) populations were conducted by EIGENSTRAT.^3^ A strict selection for SNPs with overlap with Hapmap3 SNPs was conducted: (1) MAF>0.05 and HWE>0.001; (2) Removal of 24 long LD regions (**Table I**); and (3) LD pruning with an R^2^ of 0.5; which resulted in 40,732 best quality genotyped SNPs used to calculate genetic PCs.

PCs were firstly calculated with HapMap3 population to exclude European ethnic outliers: exceeding 5 times the standard deviation of Utah residents with Northern and Western European ancestry from the CEPH collection (CEU) and Toscani in Italia (TSI) populations for the first 2 PCs (**Figure II)**. The first 2 PCs explained > 84.3% of the total. In total, 141 individuals were excluded from this dataset. Secondly, another PCA was conducted using the same SNPs (n=40,732) but calculated only in NEMESIS-2. The first 2 PCs explained >30% of the total. Another 13 individuals were considered as ethnic outliers by the first 2 PCs exceeding 5 times the standard deviation in the NEMESIS-2 sample. In the end, 3,104 individuals remained. Compared with the self-report ethnic information, 93.75% of our ethnic-QCed samples are self-reported as “Dutch”. We think some people may have understood the question as: “where were you born?”, since non-former colony countries have 0 deviations from genetic QC, while former colonies have substantial deviations. In total, 154 samples were identified as European ethnic outliers and excluded. After post-imputation quality control steps, PCA was conducted using the same SNPs (n=40,732) within NEMESIS-2 samples (n=3104). These PCs were then used as covariates in analyses to correct for population stratification.

*Figure II. First 2PCs OF NEMESIS-2 samples with Hapmap3 before exclusion of ethnic outliers.*

**

*Table I. The complex-LD regions (build GRCh37) removed for PCA analysis.*

| **Chromosome** | **Base pair start** | **Base pair end** |
| --- | --- | --- |
| 6 | 25392021 | 33392022 |
| 8 | 111930824 | 114930824 |
| 11 | 46043424 | 57243424 |
| 1 | 48287980 | 52287979 |
| 2 | 86088342 | 101041482 |
| 2 | 134666268 | 138166268 |
| 2 | 183174494 | 190174494 |
| 3 | 47524996 | 50024996 |
| 3 | 83417310 | 86917310 |
| 3 | 88917310 | 96017310 |
| 5 | 44464243 | 50464243 |
| 5 | 97972100 | 100472101 |
| 5 | 128972101 | 131972101 |
| 5 | 135472101 | 138472101 |
| 6 | 56892041 | 63942041 |
| 6 | 139958307 | 142458307 |
| 7 | 55225791 | 66555850 |
| 8 | 7962590 | 11962591 |
| 8 | 42880843 | 49837447 |
| 10 | 36959994 | 43679994 |
| 11 | 87860352 | 90860352 |
| 12 | 33108733 | 41713733 |
| 12 | 111037280 | 113537280 |
| 20 | 32536339 | 35066586 |

***2. Training schizophrenia GWAS summary statistic processing and calculating polygenic risk score for schizophrenia***

We used recent GWASs of schizophrenia^4^ for PRS calculations.^5^ As a quality control for PRS calculation, the SNPs that overlapped between the GWASs summary statistics (training datasets) and our dataset were extracted. Then, insertions or deletions, ambiguous SNPs, SNPs with minor allele frequency (MAF) <0.01 and imputation quality (R^2^) < 0.8 in both training and target datasets were excluded. To account for complicated LD structure of SNPs in the genome, these SNPs were clumped in two rounds using PLINK 1.90b3z^6^ according to previously established methods;^7,8^ round 1 with the default parameters (physical distance threshold 250kb and LD threshold (R^2^) 0.5); and round 2 with a physical distance threshold of 5,000kb and LD threshold (R^2^) 0.2. Additionally, we excluded all SNPs in genomic regions with strong or complex LD structures (e.g. the MHC region on chromosome 6; **Table I**). The odds ratios (ORs) were reported in the summary statistics and were log-converted to beta values as effect sizes. Sample overlap between NEMESIS-2 data with schizophrenia GWAS cohort (PCG and CLOZUK cohorts) is unlikely since all samples belong to different cohorts. We constructed PRS based on schizophrenia risk alleles weighted by their schizophrenia increasing effect estimate using the Purcell et al. method,^2^ i.e. using PLINK’s score function. PRS was calculated for 3104 samples (those remaining after QC). Informed by the PGC analyses, PRS for schizophrenia with a significance cut-off p<0.05 was used in the analyses to achieve a balance between the number of false-positive and true-positive risk alleles.^9^

# **Supplementary Table 1. Interactions between schizophrenia risk scores and sex**

| **Outcome** | **Trend between PRS-SCZ and outcome (95% CI)** | |
| --- | --- | --- |
|  | In male participants | In female participants |
| Mental health | -0.06 (-0.11, -0.02) | -0.07 (-0.11, -0.03) |
| Physical health | -0.00 (-0.05, 0.04) | -0.04 (-0.08, 0.00) |
| **Outcome** | **Trend between ES-SCZ and outcome (95% CI)** | |
|  | In male participants | In female participants |
| Mental health | -0.20 (-0.23, -0.17) | -0.23 (-0.25, -0.20) |
| Physical health | -0.11 (-0.14, -0.08) | -0.19 (-0.22, -0.17) |

Linear mixed models of mental health and physical health scores as measured with the 36-item Short Form Survey. PRS-SCZ indicates polygenic risk score for schizophrenia. ES-SCZ indicates exposome score for schizophrenia. All models are adjusted for time of outcome since T0. PRS-SCZ models are also adjusted for the first three principal components.

# **Supplementary Table 2. Sensitivity analysis of the interaction between ES-SCZ and age in association with mental health**

| **ES-SCZ** | **Interaction term** | | | |
| --- | --- | --- | --- | --- |
|  | Coef, with linear age (95% CI) | p-value | Coef, with quadratic age (95% CI) | p-value |
| Without cannabis use | -1.32 (-5.00, 2.37) | .48 | 4.89 (1.19, 8.60) | .01 |
| Without winter birth | -3.65 (-6.98, -0.31) | .03 | 4.01 (0.62, 7.39) | .02 |
| Without hearing impairment | -4.30 (-7.71, -0.89) | .01 | 3.40 (-0.04, 6.84) | .05 |
| Without emotional abuse | -4.79 (-8.83, -0.74) | .02 | 3.62 (-0.47, 7.72) | .08 |
| Without physical abuse | -3.50 (-6.69, -0.30) | .03 | 3.64 (0.39, 6.88) | .03 |
| Without sexual abuse | -4.45 (-8.17, -0.74) | .02 | 3.48 (-0.27, 7.23) | .07 |
| Without emotional neglect | -3.87 (-7.55, -0.19) | .04 | 3.73 (0.02, 7.45) | .05 |
| Without bullying | -5.29 (-9.95, -0.64) | .03 | 8.86 (4.11, 13.62) | .0003 |

Linear mixed models of mental health scores as measured with the 36-item Short Form Survey. ES-SCZ indicates exposome score for schizophrenia, from which were alternately excluded single exposures. All models are adjusted for time of outcome since T0.

# **Supplementary Table 3. Sensitivity analysis of the interaction between ES-SCZ and age in association with physical health**

| **ES-SCZ** | **Interaction term** | | | |
| --- | --- | --- | --- | --- |
|  | Coef, with linear age (95% CI) | p-value | Coef, with quadratic age (95% CI) | p-value |
| Without cannabis use | -3.32 (-7.13, 0.50) | .09 | 2.80 (-1.03, 6.64) | .15 |
| Without winter birth | -5.95 (-9.41, -2.49) | .0008 | 1.49 (-2.02, 5.00) | .41 |
| Without hearing impairment | -6.60 (-10.13, -3.06) | .0003 | 0.52 (-3.05, 4.10) | .77 |
| Without emotional abuse | -7.79 (-11.97, -3.62) | .0003 | 0.80 (-3.42, 5.02) | .71 |
| Without physical abuse | -5.86 (-9.18, -2.55) | .0005 | 1.15 (-2.21, 4.52) | .50 |
| Without sexual abuse | -6.94 (-10.80, -3.09) | .0004 | 1.02 (-2.86, 4.91) | .61 |
| Without emotional neglect | -6.48 (-10.28, -2.69) | .0008 | 1.58 (-2.25, 5.41) | .42 |
| Without bullying | -7.79 (-12.61, -2.97) | .002 | 3.70 (-1.23, 8.63) | .14 |

Linear mixed models of physical health scores as measured with the 36-item Short Form Survey. ES-SCZ indicates exposome score for schizophrenia, from which were alternately excluded single exposures. All models are adjusted for time of outcome since T0.

# **Supplementary Table 4: Sensitivity analysis of the interaction between ES-SCZ and sex in association with physical health**

| **ES-SCZ** | **Interaction coefficient (95% CI)** | **p-value** | **Trend in male participants (95% CI)** | **Trend in female participants (95% CI)** |
| --- | --- | --- | --- | --- |
| Without cannabis use | -0.08 (-0.13, -0.02) | .01 | -0.18 (-0.23, -0.14) | -0.26 (-0.29, -0.23) |
| Without winter birth | -0.10 (-0.15, -0.05) | <.0001 | -0.13 (-0.17, -0.09) | -0.24 (-0.27, -0.20) |
| Without hearing impairment | -0.11 (-0.16, -0.06) | <.0001 | -0.13 (-0.17, -0.09) | -0.24 (-0.27, -0.20) |
| Without emotional abuse | -0.13 (-0.19, -0.07) | <.0001 | -0.13 (-0.17, -0.08) | -0.26 (-0.30, -0.22) |
| Without physical abuse | -0.10 (-0.14, -0.05) | <.0001 | -0.14 (-0.17, -0.10) | -0.23 (-0.26, -0.20) |
| Without sexual abuse | -0.11 (-0.17, -0.06) | <.0001 | -0.13 (-0.17, -0.09) | -0.24 (-0.28, -0.21) |
| Without emotional neglect | -0.11 (-0.17, -0.06) | <.0001 | -0.13 (-0.17, -0.09) | -0.24 (-0.28, -0.21) |
| Without bullying | -0.15 (-0.22, -0.08) | <.0001 | -0.14 (-0.19, -0.08) | -0.29 (-0.34, -0.24) |

Linear mixed models of physical health scores as measured with the 36-item Short Form Survey. ES-SCZ indicates exposome score for schizophrenia, from which were alternately excluded single exposures. All models are adjusted for time of outcome since T0.

# **Supplementary Figure 1. Sensitivity analysis of the interaction between ES-SCZ and age in association with mental health**


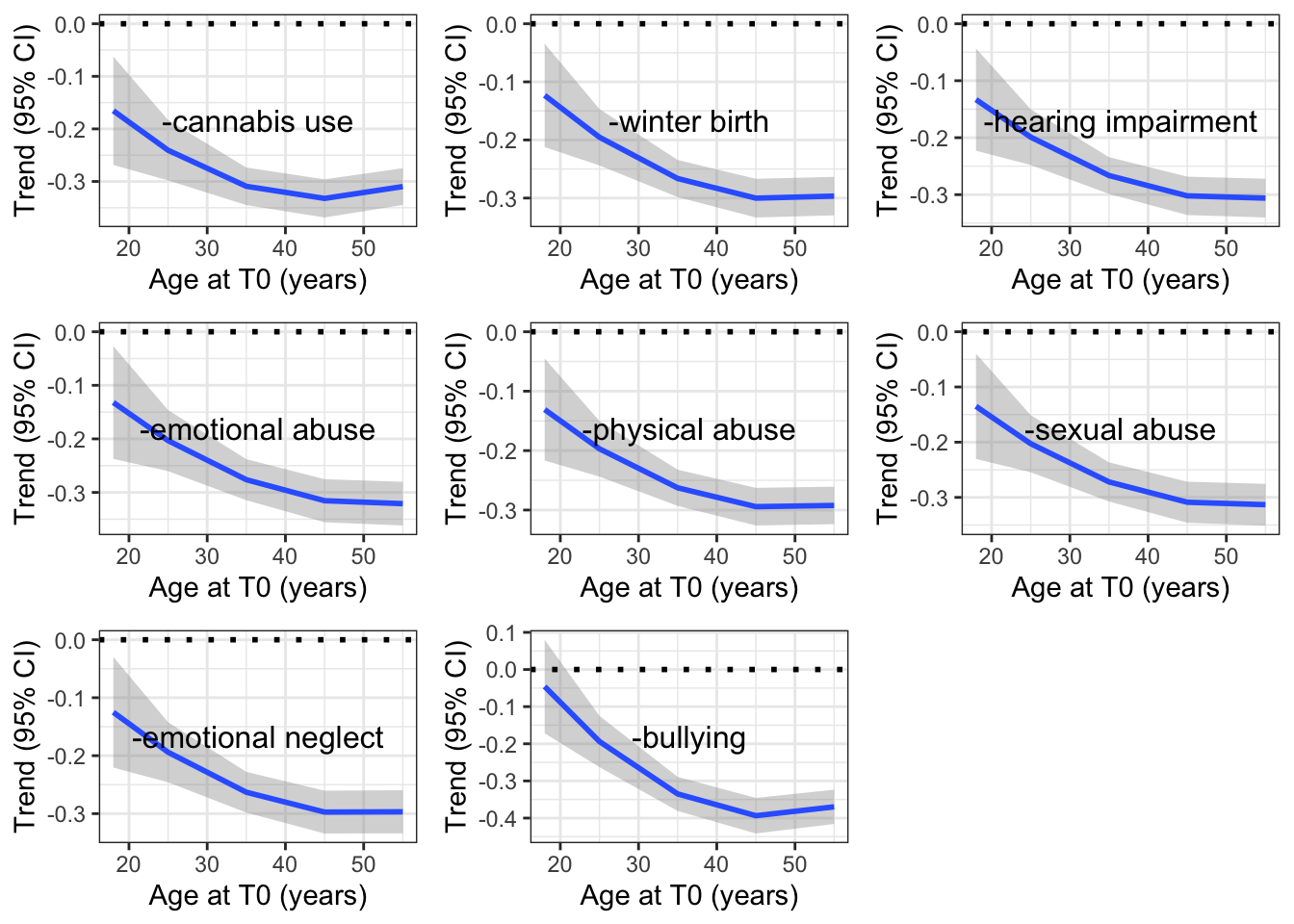


ES-SCZ indicates exposome score for schizophrenia, from which were alternately omitted single exposures, which are indicated on each graph. Using linear mixed models, we estimated the trends between variations of ES-SCZ and mental health according to age, including linear and quadratic effects of age on these trends.

# **Supplementary Figure 2. Sensitivity analysis of the interaction between ES-SCZ and age in association with physical health**


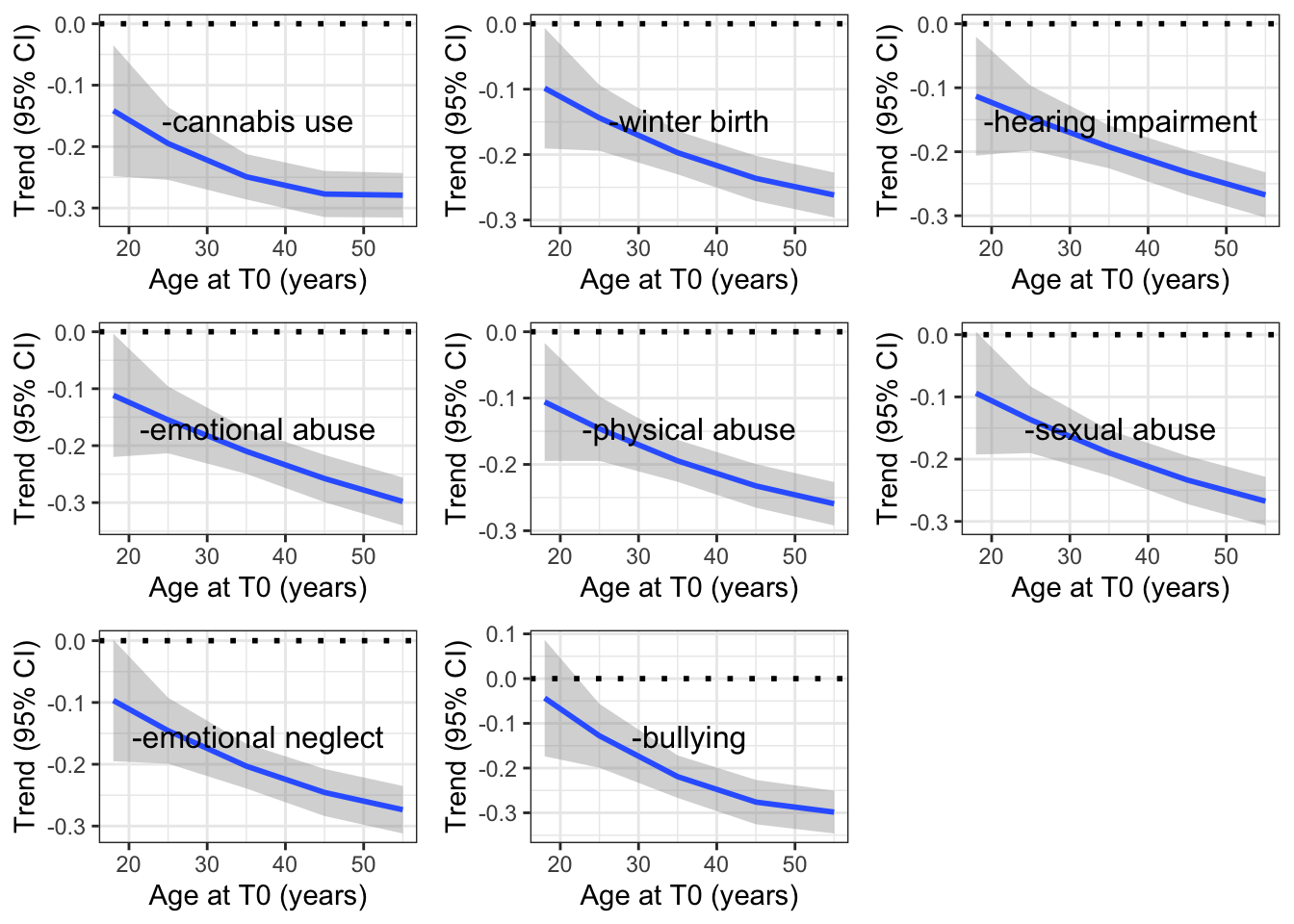


ES-SCZ indicates exposome score for schizophrenia, from which were alternately omitted single exposures, which are indicated on each graph. Using linear mixed models, we estimated the trends between variations of ES-SCZ and physical health according to age, including linear and quadratic effects of age on these trends.

# **References**

1. European Network of National Networks studying Gene-Environment Interactions in Schizophrenia (EU-GEI). Identifying Gene-Environment Interactions in Schizophrenia: Contemporary Challenges for Integrated, Large-scale Investigations. *Schizophrenia Bulletin*. 2014;40(4):729-736. doi:10.1093/schbul/sbu069

2. Purcell S, Neale B, Todd-Brown K, et al. PLINK: a tool set for whole-genome association and population-based linkage analyses. *Am J Hum Genet*. 2007;81(3):559-575. doi:10.1086/519795

3. Price AL, Patterson NJ, Plenge RM, Weinblatt ME, Shadick NA, Reich D. Principal components analysis corrects for stratification in genome-wide association studies. *Nat Genet*. 2006;38(8):904-909. doi:10.1038/ng1847

4. Pardiñas AF, Holmans P, Pocklington AJ, et al. Common schizophrenia alleles are enriched in mutation-intolerant genes and in regions under strong background selection. *Nat Genet*. 2018;50(3):381-389. doi:10.1038/s41588-018-0059-2

5. Choi SW, Mak TSH, O’Reilly PF. Tutorial: a guide to performing polygenic risk score analyses. *Nat Protoc*. 2020;15(9):2759-2772. doi:10.1038/s41596-020-0353-1

6. Chang CC, Chow CC, Tellier LC, Vattikuti S, Purcell SM, Lee JJ. Second-generation PLINK: rising to the challenge of larger and richer datasets. *Gigascience*. 2015;4:7. doi:10.1186/s13742-015-0047-8

7. Schür RR, Schijven D, Boks MP, et al. The effect of genetic vulnerability and military deployment on the development of post-traumatic stress disorder and depressive symptoms. *Eur Neuropsychopharmacol*. 2019;29(3):405-415. doi:10.1016/j.euroneuro.2018.12.009

8. McLaughlin RL, Schijven D, van Rheenen W, et al. Genetic correlation between amyotrophic lateral sclerosis and schizophrenia. *Nat Commun*. 2017;8(1):14774. doi:10.1038/ncomms14774

9. Wray NR, Lee SH, Mehta D, Vinkhuyzen AAE, Dudbridge F, Middeldorp CM. Research review: Polygenic methods and their application to psychiatric traits. *J Child Psychol Psychiatry*. 2014;55(10):1068-1087. doi:10.1111/jcpp.12295
